# *Flos Lonicera* Ameliorates Obesity and Associated Endotoxemia in Rats through Modulation of Gut Permeability and Intestinal Microbiota

**DOI:** 10.1371/journal.pone.0086117

**Published:** 2014-01-24

**Authors:** Jing-Hua Wang, Shambhunath Bose, Gi-Cheol Kim, Seung-Ug Hong, Ji-Hun Kim, Jai-eun Kim, Hojun Kim

**Affiliations:** 1 Department of Oriental Rehabilitation Medicine, Dongguk University, Goyang, Gyeonggi-do, Republic of Korea; 2 Key Laboratory of Xin’an Medicine, Ministry of Education, Anhui University of Traditional Chinese Medicine, Hefei, Anhui Province, People’s Republic of China; 3 College of Pharmacy, Dongguk University-Seoul, Goyang, Gyeonggi-do, Republic of Korea; 4 Department of Oriental Otorhinolaryngology, Dongguk University, Goyang, Gyeonggi-do, Republic of Korea; 5 Department of Pathology, College of Oriental Medicine, Dongguk University, Goyang, Gyeonggi-do, Republic of Korea; National Institute of Agronomic Research, France

## Abstract

**Background and Aim:**

Increasing evidence has indicated a close association of host-gut flora metabolic interaction with obesity. *Flos Lonicera,* a traditional herbal medicine, is used widely in eastern Asia for the treatment of various disorders. The aim of this study was to evaluate whether unfermented or fermented formulations of *Flos Lonicera* could exert a beneficial impact to combat obesity and related metabolic endotoxemia.

**Methods:**

Obesity and metabolic endotoxemia were induced separately or together in rats through feeding a eight-week high fat diet either alone (HFD control group) or in combination with a single LPS stimulation (intraperitoneal injection, 0.75 mg/kg) (LPS control group). While, the mechanism of action of the *Lonicera* formulations was explored *in vitro* using RAW 264.7 and HCT 116 cell lines as models.

**Results:**

In cell-based studies, treatment with both unfermented *Flos Lonicera* (UFL) and fermented *Flos Lonicera* (FFL) formulations resulted in suppression of LPS-induced NO production and gene expression of vital proinflammatory cytokines (TNF-α, COX-2, and IL-6) in RAW 264.7 cells, reduced the gene expression of zonula occludens (ZO)-1 and claudin-1, and normalized trans epithelial electric resistance (TEER) and horseradish peroxidase (HRP) flux in LPS-treated HCT-116 cells. In an animal study, treatment of HFD as well as HFD+LPS groups with UFL or FFL resulted in a notable decrease in body and adipose tissue weights, ameliorated total cholesterol, HDL, triglyceride, aspartate transaminase and endotoxin levels in serum, reduced the urinary lactulose/mannitol ratio, and markedly alleviated lipid accumulation in liver. In addition, exposure of HFD as well as HFD+LPS groups with UFL or FFL resulted in significant alteration of the distribution of intestinal flora, especially affecting the population of *Akkermansia* spp. and ratio of Bacteroidetes and Firmicutes.

**Conclusion:**

This evidence collectively demonstrates that *Flos Lonicera* ameliorates obesity and related metabolic endotoxemia via regulating distribution of gut flora and gut permeability.

## Introduction

Obesity, which was officially declared a disease by the World Health Organization (WHO) in 1997 [1], is a serious and alarming global public health issue as it can evidently increase the risk of hyperlipidemia, hypertension, diabetes, coronary-heart diseases, and certain cancers [2]. According to the predicted statement of WHO, approximately 2.3 billion adults worldwide will be overweight by 2015 and 700 million of them will be obese [3]. Although overweight and obesity are generally regarded as the consequences of an imbalance in energy homeostasis, on the basis of accumulating evidence, obesity is now recognized as a chronic and systemic inflammatory disease, where adipose tissue plays a vital endocrine role [4]. Local inflammatory loops in adipose tissue have been reported to become operational as a consequence of nutrient overload. Accordingly, crosstalk among the cellular constituents of adipose tissue, such as adipocytes, and endothelial and immune cells leads to augmentation of inflammatory mediators. These mediators exert important systemic effects that can result in development of insulin resistance, dysmetabolism, and cardiovascular disease [4].

Besides, accumulating evidence has also suggested that host-gut microbial metabolic interactions can play an important role in predisposing to obesity [5]–[7], which is mediated via an increase in the capacity of energy harvest from a given diet and induction of low-grade chronic systemic inflammation [8]–[11]. Some studies have demonstrated that increased uptake of gut microbiota-derived endotoxin/lipopolysaccharide (LPS) in mice fed a high fat diet (HFD) could lead to metabolic endotoxemia, which in turn may trigger obesity and insulin resistance [11], [12]. However, quite interestingly, the LPS-mediated pathological conditions described above were ameliorated when the gut microbial homeostasis was modulated by prebiotic and antibiotic treatments [12], [13].

Accordingly, modulation of the gut microbiota distribution by probiotics/prebiotics is deemed a more effective and feasible strategy for control of obesity and its associated metabolic syndrome [14]–[16]. A number of herbal products have been reported to exert beneficial effects against obesity and anti-inflammation in both animal and human subjects; nevertheless, few studies have systemically assessed the relationship between herbal medicine and gut flora in an obesity/endotoxemia model in detail [17]–[19].

*Lonicera japonica* Thunb., a medicinal and edible herb, which originated from eastern Asia, has been widely used as a traditional treatment for many diseases for a long time. This herb contains a variety of compounds, including organic acids, flavonoids, iridoid glycosides, and saponins etc. [20], and possesses many beneficial pharmacological properties including antiinflammatory [21], [22], antioxidative [23], hepatoprotective [24], antihypertensive [25], antinociceptive [22], and antiviral [26], [27] activities.

Fermentation is frequently applied for degradation or conversion of certain undesirable substrate components into compatible ones; this process can also modulate the pharmacological activities of biological substrates primarily through modification of naturally occurring molecules such as isoflavones, saponins, phytosterols, and phenols. The beneficial impact on health and the anti-inflammatory activities of fermented herbal or food products, either alone or in combination, are well documented [28]–[33]. In addition, probiotics used for fermentation may also exert health-promoting effects [32], [34], and the anti-inflammatory properties of probiotics have also been reported by a number of studies [32], [35].

Despite the beneficial health effects of *Flos Lonicera* as mentioned above, no scientific studies have been conducted so far to judge whether this herb can protect against obesity and associated disorders. This prompted us to evaluate the pharmacological effects of *Flos Lonicera* against high fat diet-induced obesity and accompanying endotoxin insult, and to determine whether this beneficial impact is further boosted by fermentation of the herb along with use of probiotic involved in fermentation. In addition, the anti-inflammatory activities and impact of *Flos Lonicera* on the distribution profile of intestinal microbiota and gut permeability were also assessed in order to elucidate the possible mechanism(s) by which this herb may exert its protective effects against obesity and associated endotoxemia.

## Materials and Methods

### Microbiological Culture

The stock inoculum of *Lactobacillus plantarum* (Cell Biotech Co., Gimpo, South Korea) was cultured at 37°C for 24 h twice in Lactobacilli MRS broth (Difco-BD, Sparks, MD, USA) under aseptic conditions and finally grown in the same broth prior to use in the fermentation process.

### Extraction and Fermentation of *Flos Lonicera*

*Flos Lonicera* (The Korean Pharmacopoeia standard grade) was purchased from the Medical Supply Store of Dongguk International Hospital (Goyang, South Korea). The identity of this herb was further confirmed by Prof. Je-Hyun Lee (College of Oriental Medicine, Dongguk University, South Korea). Extraction and fermentation of *Flos Lonicera* were performed as described previously [28]. Briefly, dried powder (30 g) of *Flos Lonicera* was dissolved in 250 ml of distilled water and mixed vigorously. The preparation was subjected to ultrasonication for 30 min at room temperature (RT), followed by incubation at 70°C for 3 h in a shaking incubator. The product obtained was autoclaved for 15 min at 121°C for sterilization and decoction. Subsequently, the herbal extract was inoculated with a fresh subculture of *Lactobacillus plantarum* to yield the final bacterial count of 2×10^7^ colony forming units (CFU)/ml. The resultant mixture was incubated for 24 h at 37°C to produce the fermented *Flos Lonicera* (FFL) where the starter bacterial population was kept alive further in order to achieve the probiotic effects. The unfermented *Flos Lonicera* (UFL) was prepared in a similar way, except that it received 2% (v/v) of the sterile Lactobacilli MRS broth instead of the bacterial inoculum. Finally, the extracted preparations were subjected to low speed centrifugation in order to sediment the particles and the resultant supernatants were freeze dried at −50°C for 72 h. The yielded products were stored at −70°C for future use.

### Fingerprinting Analysis

HPLC-based fingerprinting of the UFL and FFL preparations was performed using a binary HPLC system (Flexar, Perkin Elmer, CA, USA) equipped with a binary pump, an autosampler/injector, a column oven, and a UV/visible detector. For analyses of the samples, two major components of *Flos Lonicera* (Chlorogenic acid and quercetin, Sigma-Aldrich, MO, USA) were used as standards. The standards were dissolved in 0.4% phosphoric acid (Junsei, Tokyo, Japan) with the final concentration of chlorogenic acid at 25 µg/ml and quercetin at 20 µg/ml, while UFL and FFL were dissolved separately in the same solvent at a final concentration of 100 µg/ml. After filtration through a 0.45 µm membrane filter (Sartorius AG, Goettingen, Germany), 20 µl aliquots of the standard or extracted samples were injected into the HPLC system via an autosampler. The gradient separation of samples was performed on an Agilent Eclipse XDB-C18 column (5 µm, 250×4.6 mm) at 30°C. The mobile phase was composed of 0.4% phosphoric acid (A) and 99.9% acetonitrile (Merck, Darmstadt, Germany) (B) and the linear gradient was maintained at 95, 87, 70, 60, and 95% of A at 0, 8, 20, 35, and 45 min, respectively. The elution was performed at a flow rate of 1.0 ml/min and the detection was performed at 238 nm. Chromera manager software (Perkin Elmer) was utilized for control of the system, as well as data acquisition and analyses.

### Cell Culture

In order to understand the molecular mechanism underlying the beneficial impact of *Flos Lonicera*, RAW 264.7 (Murine-leukemic monocyte-macrophage) and HCT-116 (colorectal carcinoma epithelial cells) cell lines (Korea Cell Line Bank, Seoul, Korea) were used in performance of *in vitro* experiments. RAW 264.7 cells were cultured in Dulbecco’s Modified Eagle Medium (DMEM, GIBCO, USA) supplemented with 10% fetal bovine serum (FBS, GIBCO, USA origin). HCT-116 cells were grown in McCoy’s 5A medium (modified, Invitrogen Carlsbad, CA, USA) containing 10% FBS, 100 U/mL penicillin, and 100 µg/mL streptomycin. Both cell lines were cultured in an incubator at 37°C under a humidified environment of air containing 5% CO_2_.

### Measurement of Nitric Oxide (NO) Production by RAW 264.7 Cells

The *in vitro* anti-inflammatory activity of UFL or FFL was evaluated in terms of their ability to inhibit NO production in LPS-induced-RAW 264.7 cells. Briefly, after three to four cycles of subculturing, RAW 264.7 cells were seeded at a density of 2×10^5^ cells/well in 24-well plates. Following overnight growth, the cells were treated with DMEM, UFL or FFL (100, 200, or 400 µg/ml) for 4 h, followed by addition of lipopolysaccharide (LPS, from *Pseudomonas aeruginosa*, Sigma-Aldrich, final concentration of 0.2 µg/ml, pH 7.4) or DMEM, respectively, for 24 h. The nitrite production content in the supernatant was determined using the Griess reagent system kit (Promega, WI, USA) in accordance with the instructions of the kit manufacturer.

### Determination of Gene Expression of Proinflammatory Cytokines and Mediators in RAW 264.7 Cells and Tight Junction Proteins in HCT 116 Cells by Real-time PCR

In order to elucidate the molecular mode of action of UFL and FFL on the inflammatory pathway, the effects of these formulations on the gene expression of IL-6, tumor necrosis factor alpha (TNF-α), and cyclooxygenase 2 (COX-2) were examined in LPS-induced RAW 264.7 cells. Briefly, after three to four cycles of subculturing, RAW 264.7 cells were adjusted to 1×10^7^ per well in 6-well plates. After treatment with DMEM, UFL or FFL (100, 200, or 400 µg/ml) for 4 h, LPS (0.2 µg/ml) or DMEM was added, respectively, for 4 h. Similarly, In order to evaluate the impact of UFL and FFL on the regulatory proteins of gut permeability, the effect of these drugs on the gene expression of the two vital tight junction proteins (TJP) namely, claudin-1 and zonula occludens-1 (ZO-1), were examined in HCT-116 cells. Briefly, after treatment with McCoy’s 5A medium, UFL or FFL (100, 200, or 400 µg/ml) for 2 h in HCT-116 cells (1×10^7^/well), LPS (0.2 µg/ml) or McCoy’s 5A medium was treated, respectively, for 6 h.

Following this, total RNA from LPS-stimulated RAW 264.7 or HCT-116 cells was isolated using TRIsure reagent (Bioline, MA, USA), followed by synthesis of cDNA using the AccuPower RT premix kit (Bioneer, Daejeon, Korea) according to the instructions of the kit manufacturer. The standard conditions for the PCR amplification reactions were applied using the listed primers (Table 1) in accordance with our previous method [29]. The LightCycler software (Roche Applied Science) was used for analyzing. Quantification of relative genes expression was represented by 2^–ΔCt^ calculations (Δ*C*_t_ =  *C*_t_-_target gene_ − *C*_t -GAPDH_) and then normalized by GAPDH.

**Table 1:** Primer sequences used for gene expression analysis by real-time PCR.

Gene name	Primer sequence	OAT	Product size	
COX-2	5′-AGA AGG AAA TGG CTG CAG AA-3′	53°C	194 bp	
	5′-GCT CGG CTT CCA GTA TTG AG-3′			
IL-6	5′-AGT TGC CTT CTT GGG ACT GA-3′	60°C	190 bp	
	5′-CAG AAT TGC CAT TGC ACA AC-3′			
TNF-α	5′-GAA CTG GCA GAA GAG GCA CT-3′	60°C	203 bp	
	5′-AGG GTC TGG GCC ATA GAA CT-3′			
ZO-1	5′-CCA GTC CCT TAC CTT TC-3′	52°C	93 bp	
	5′-CTC CTC CAG TCT GAC ATT AG-3′			
claudin-1	5′- CTG TTG GGC TTC ATT CTC G-3′	52°C	109 bp	
	5′- GGG CGG TCA CGA TGT TGT-3′			
GAPDH	5′-TGA TGA CAT CAA GAA GGTGGT GAA G-3′	56°C	240 bp	
	5′-TCC TTG GAG GCC ATG TAG GCC AT-3′			

Abbreviations: OAT, optimized annealing temperature; COX-2, cyclooxygenase 2; IL-6, interlukin 6; TNF-α, tumor necrosis factor alpha; ZO-1, zonula occludens-1; GAPDH, glyceraldehydes-3-phosphate dehydrogenase.

### Determination of Transepithelial Electrical Residence (TEER) and Horseradish Peroxidase (HRP) Flux in HCT-116 Cells

These assays were performed as described previously [30] with some modifications. After three to four cycles of subculturing, HCT-116 cells were adjusted to 2×10^5^ per well onto the apical wells of Millicell-24 cell culture insert plates (12 mm in diameter 0.4 µm membrane pore size; Millipore, Bedford, MA, USA) and grown as monolayers. After treatment with different concentrations of UFL or FFL (0, 100, 200, or 400 µg/ml) for 24 h, HCT-116 cells were treated with LPS (10 µg/ml) or McCoy’s medium. After LPS treatment for 20 h, TEER on the inside and outside of the apical wells was measured using a Millicell ERS-2 epithelial volt-ohm meter (Millipore, USA) according to the instructions of the manufacturer. The results were expressed as a percentage of the normal.

For the HRP flux assay, HCT-116 cells were treated in the manner described above for TEER measurements, except that the duration of LPS treatment was maintained for 24 h instead of 20 h. The cells were then washed with Dulbecco’s Phosphate-Buffered Saline (DPBS, GIBCO, CA, USA). Only apical compartments were filled with 360 µl of DPBS. HRP (Sigma-Aldrich, MO, USA; final concentration is 0.01 mg/ml) was added to apical wells for incubation in a 37°C at 5% CO_2_ environment for 1 h. Finally, 1 *μ*l of penetrating fluid from the apical to the basolateral compartment was mixed with 100 *μ*l of 3, 3′, 5, 5′-tetramethylbenzidine (TMB; Sigma-Aldrich, MO, USA) as the substrate. After addition of 100 *μ*l of stop solution (2 M H_2_SO_4_), the absorbance was measured at 450 nm using a spectrophotometer (Spectramax plus, Molecular Devices, USA). The results of HRP flux were expressed as a percentage of the normal.

### Animals and Experimental Design

Male Sprague-Dawley (SD) rats (180–220 g) were purchased from Orient Bio (Seongnam, Korea). Prior to performance of the experiment, animals were acclimatized for seven days at a controlled temperature (20±2°C) with relative humidity of 40–60% and a 12 h light-dark cycle with a commercial normal chow diet (Soyagreentec, Hwaseong, Korea) and tap water *ad libitum*. Forty eight rats were then randomly divided into six groups of eight rats each, as follows: normal, HFD control (high fat diet only), LPS control (HFD +0.75 mg/kg of LPS), UFL (250 mg/kg of UFL+HFD +0.75 mg/kg of LPS), FFL (250 mg/kg of FFL+HFD +0.75 mg/kg of LPS), and colostrum control (10% of colostrum+HFD +0.75 mg/kg of LPS). The colostrum treatment was chosen as a positive control in our experiment because colostrum belongs to an over-the-counter natural prebiotics which is constituted by abundant bioactive compounds possessing multifounctional bioactivity, including anti-oxidative, anti-inflammatory and immunoregulatory activities [36]–[38]. Besides, colostrum plays an important role in maintaining intestinal homeostasis which is mediated through the regulation of intestinal permeability [39], [40]. It has been demonstrated that bovine colostrum might combat inflammation of intestinal epithelial cells by inhibiting the NF-κB pathway [41]. In our previous studies evaluating the *in vitro* and *in vivo* anti-inflammatory activities of herbal formulations, colostrum was used as a positive control in the animal studies [29], [30]. Moreover, it was shown that the obesity-related parameters like total cholesterol and triglyceride levels were decreased in Type 2 diabetic patients following the ingestion bovine colostrum [42].

Rats in the normal group were given the normal diet continuously for a period of eight weeks. Commercial high fat diet (Soyagreentec, Hwaseong, Korea) was fed to all groups, except the normal group, with the same schedule as the normal group. At the onset of sixth week, UFL (250 mg/kg), FFL (250 mg/kg), and colostrum (1 ml/rat, 10% of a commercial product, Colostrum technologies GmbH, Germany) were administered by oral gavage once per day for a period of three weeks separately in the corresponding group. Distilled water was given instead of herbal drugs in the normal and HFD control group. At the end of the eighth week, intraperitoneal injection of LPS (0.75 mg/kg) was administered only once to all groups, except the normal and HFD control group. After LPS injection for 12 h, the animals of all groups were transferred to individual metabolic cages and were fasted for 12 h with water *ad libitum*. Subsequently, 1 ml of mixture solution (containing 66 mg/ml lactulose and 50 mg/ml mannitol) was administered by oral gavage. After another 20 h fast, stool and urine samples were collected and stored at −70°C, and all animals were then weighed and sacrificed. Blood was collected from the abdominal aorta under ether anesthesia. After weighing the abdominal and epididymal fat pads, the portions of liver tissue were removed and stored at −70°C for future use.

The animal experiment was performed according to the international guidelines (Guide for the Care and Use of Laboratory Animals, Institute of Laboratory Animal Resources, Commission on Life Sciences, National Research Council, USA; National Academy Press: Washington D.C., 1996). The design and protocols for the animal experiment were approved by the Institutional Animal Ethical Committee of Dongguk University (Permit Number: 2011-1268). All surgery was performed under Zoletil® (tiletamine-zolazepam, Virbac, Carros, France) and Rompun® (xylazine-hydrochloride, Bayer, Leverkusen, Germany) combination anesthesia, and all efforts were made to minimize suffering.

### Lipid Deposition Findings in Liver Tissue by Oil Red O staining

Frozen liver tissues embedded in FSC 22 frozen section compound (Leica, USA) were sectioned at 8 mm, mounted on silicone coated slides (Leica, USA), and then stained with oil red O solution (Cayman chemical, USA) for 10 min at 60°C and Mayer’s hematoxylin solution for 1 min. All of the slides were observed under an Olympus BX61 microscope (Tokyo, Japan) and photographed using an Olympus DP70 digital camera (Tokyo, Japan).

### Determination of Lactulose and Mannitol in Urine

To examine the intestinal passive permeability, urinary lactulose and mannitol were measured using K-Lactul and K-Manol kits (Megazyme, Wicklow, Ireland) according to the manufacturer’s instructions. The final results were expressed as a ratio of lactulose to mannitol (L/M).

### Biochemical Analysis in Serum

Rat serum was separated using Vacutainer tubes (BD, Plymouth, UK) after 1 h of blood clotting. The serum levels of total cholesterol, HDL, triglyceride (TG), and aspartate transaminase (AST) were determined using commercial kits from Asan Pharmaceutical (Seoul, Korea). The levels of serum endotoxin were determined using a Limulus Amebocyte Lysate (LAL) kit (ENDOSAFE, SC, USA) according to the manufacturer’s protocol.

### Determination of Proinflammatory Cytokines in Serum by ELISA

The rat serum levels of TNF-α and C-reactive protein (CRP) were measured using commercial rat-specific ELISA kits according to the manufacturers’ instructions (BioLegend, San Diego, CA, USA; USCN, Huston, TX, USA).

### Microbial Analysis of Rat Stool by PCR-DGGE (Denaturing Gradient Gel Electrophoresis) and Real-time PCR

Before sacrifice, fresh rat stools were collected and stored at −70°C. The genomic DNA from the stool was isolated using an AccuPrep stool DNA extraction kit (Bioneer, Daejeon, Korea) for analysis of the profile and quantitative composition of the intestinal microbial community.

The stool genomic DNA and universal bacterial primer for 16s rRNA genes (5′-AGA GTT TGA TCC TGG CTC AG-3′ and 5′-AAG GAG GTG ATC CAG CC-3′) was used for routine performance of the PCR procedure. After electrophoresis of the first PCR products in 1% agarose gel and staining with ethidium bromide (EB, Bio-Rad, CA, USA), only 1.5 kb bands of gels were cut and DNA was then extracted using an Accuprep gel purification kit (Bioneer, Daejeon, Korea). The second round of PCR amplified the V3 region from 16S rDNA using the primer with a GC-clamp (314f-GC: 5′-CGC CCG CCG CGC GCG GCG GGC GGG GCG GGG GCA CGG GGG GCC TAC GGG AGG CAG CAG-3′ and 518r: 5′-ATT ACC GCG GCT GCT GG-3′). Subsequently, DGGE was performed using the DCode universal mutation detection system (Bio-Rad, CA, USA). The second PCR products (20 µl) were loaded into 40% Acrylamide/bis (37.5∶1) gels with denaturing gradients from 30–60% (where 100% is 7 M urea and 40% (v/v) formamide) in 1×TAE buffer (ViroMed, Seoul, Korea). Electrophoresis was performed at 80 V at a temperature of 60°C for 15 h. After staining with EB at RT for 15 min, the gels were photographed under UV illumination (LAS-3000; Fuji photo film, Tokyo, Japan). Analysis of the DGGE patterns was performed using Bionumerics 3.0 software (Applied Maths, Sint-Martens-Latem, Belgium). All of the gels were analyzed using an unweighted pair group method, using an arithmetic means (UPGMA) clustering procedure based on genetic similarity expressed by the Jaccard coefficient. In addition, principal component analysis (PCA) was also performed on the DGGE profiles.

Real-time PCR was performed using a lightCycler FastStart DNA Master SYBR Green kit and a LightCycler instrument (Roche Applied Science, Indianapolis, ID, USA). The listed primer sequences were used in targeting the 16S rRNA gene of the bacteria (Table 2). The standard conditions for the PCR amplification reactions were applied, as previously described [28]. The LightCycler software (Roche Applied Science) was used for analyzing. Quantification of bacterial abundance was represented by 2^–Ct^ calculations. The final results are expressed as normalized fold values relative to the normal group.

**Table 2:** Primer sequences used for gut microbiota analysis by real-time PCR.

Gene name	Primer sequence	OAT	Ref.
*Lactobacillus* spp.	5′-GAG GCA GCA GTA GGG AAT CTT C-3′	60°C	[12]
	5′-GGC CAG TTA CTA CCT CTA TCC TTC TTC-3′		
*Bifdobacterium* spp.	5′-CGC GTC TGG TGT CAA AG-3′	65°C	[71]
	5′-CCC CAC ATC CAG CAT CCA-3′		
*Ruminococcus* spp.	5′-GGC GGC CTA CTG GGC TTT-3′	60°C	[72]
	5′-CCA GGT GGA TAA CTT ATT GTG TTA A-3′		
*Akkermansia* spp.	5′-CAG CAC GTG AAG GTG GGG AC-3′	60°C	[10]
	5′-CCT TGC GGT TGG CTT CAG AT-3′		
Bacteroidetes	5′-GGA RCA TGT GGT TTA ATT CGA TGA T-3′	66°C	[73]
	5′-AGC TGA CGA CAA CCA TGC AG-3′		
Firmicutes	5′-GGA GYA TGT GGT TTA ATT CGA AGC A-3′	69°C	[73]
	5′-AGC TGA CGA CAA CCA TGC AC-3′		

Abbreviations: OAT, optimized annealing temperature; Ref., reference.

### Statistical Analysis

The results are expressed as the mean ± standard deviation (SD). The statistical package for science software (SPSS, 17.0 version, Chicago, IL, USA) was used for analyses of all data. Statistical significance of difference was analyzed using one way analysis of variance (ANOVA) followed by the Fisher’s Least Significant Difference post-hoc test. Relationship strength between parameters was assessed using the two tailed Pearson’s correlation test. Absolute value of Pearson’s correlation coefficient r >0.4 was considered significant correlation. The difference was considered to indicate statistical significance at *P<*0.05.

## Results

### UFL and FFL Suppressed LPS-induced NO Production and Gene Expression of Vital Proinflammatory Cytokines in RAW 264.7 Cells

**Figure 1:**
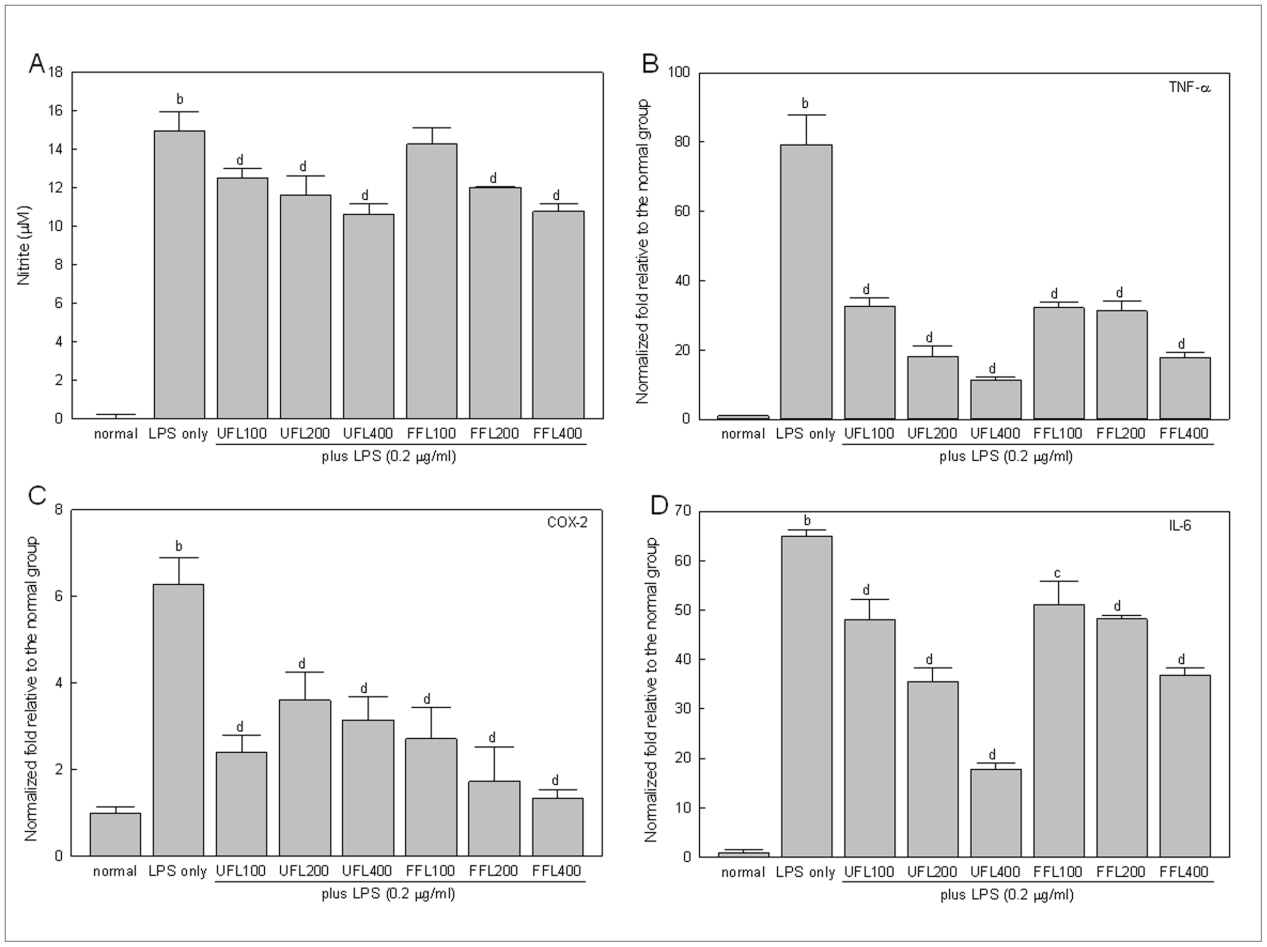
The *in vitro* anti-inflammatory activities of UFL and FFL in terms of their ability to suppress the LPS-induced production of NO and proinflammatory cytokines. (A) Immediately after pretreatment with UFL or FFL at 0 (normal, received DMEM instead of herbal extraxt), 100, 200, 400 µg/ml doses for 4 h, RAW 264.7 cells were exposed to DMEM (normal and LPS-alone) or 0.2 µg/ml LPS for 24 h following which NO measurement was performed as described in Materials and Methods section. ^b^ *P*<0.01 compared to the normal and^ c^ *P*<0.05, ^d^ *P*<0.01 compared to the LPS alone. (B–D) Immediately after pretreatment with UFL or FFL at 0 (normal, received DMEM instead of herbal extraxt), 100, 200, 400 µg/ml doses for 4 h, RAW 264.7 cells were exposed to DMEM (normal and LPS-alone) or 0.2 µg/ml LPS for 24 h following which the gene expression profile of proinflammatory cytokines (TNF-α, COX-2 and IL-6) were determined as described in Materials and Methods section. The results are expressed as normalized fold values relative to the normal.^ b^ *P*<0.01 compared to the normal and^ c^ *P*<0.05, ^d^ *P*<0.01 compared to the LPS alone.

Treatment with LPS resulted in a pronounced increase in NO production by RAW 264.7 cells (338 fold, *P*<0.01 versus normal group) (Fig. 1A). However, co-treatment of LPS-induced cells with both UFL and FFL resulted in significant, concentration-dependent inhibition of NO production (*P*<0.01). As expected, treatment with LPS caused a marked increase in transcription of TNF-α, COX-2, and IL-6 genes (*P*<0.01 versus normal). However, the expression levels of these genes in LPS-treated cells were significantly (*P*<0.01) down-regulated by both UFL and FFL in a concentration-dependent manner (Fig. 1B, C and D).

### UFL and FFL Ameliorated Endotoxemia Induced by HFD Alone or in Combination with LPS

**Figure 2:**
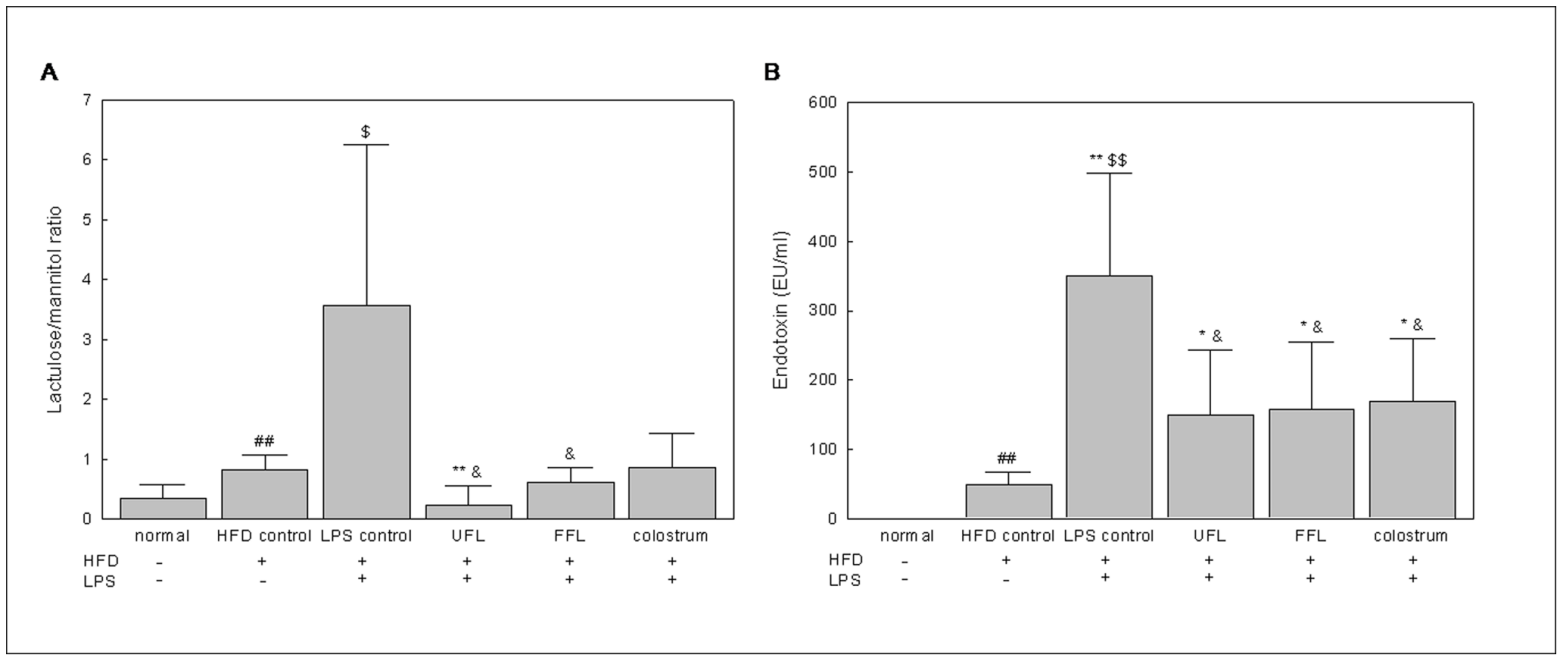
UFL and FFL attenuate HFD+LPS-induced increase in gut permeability and serum endotoxin level. (A) Following the termination of treatment schedule, the animals were fasted for 12 h with access to water *ad libitum*. Subsequently, 1.0 ml of lactulose-mannitol solution (containing 66 mg/ml lactulose and 50 mg/ml mannitol) was administered to the animals by oral gavage. After another 20 h of fasting, the urine samples were collected to determine the level of lactulose and mannitol as described in Materials and Methods section. The data are expressed as a ratio of lactulose to mannitol. (B) After the termination of experimental schedule, the blood was collected from the animals following which the serum endotoxin level was determined as described in Materials and Methods section. ^##^ *P*<0.01, compared to the normal group; ^*^ *P*<0.05, ^**^ *P*<0.01 compared to the HFD control group; ^$^ *P*<0.05, ^$$^ *P*<0.01, compared to the normal group; ^&^ *P*<0.05 compared to the LPS control group (n = 8).

Feeding of rats with HFD (HFD control group) caused a significant increase in the serum endotoxin level (*P<*0.01 versus normal group). As expected, LPS treatment in HFD-fed animals (LPS control group) further increased the level of serum endotoxin in a significant manner (*P<*0.01) (Fig. 2B), thus predisposing the animals of this group to produce a more intense inflammatory response compared to animals of the HFD group. However, co-exposure of the HFD+LPS group to either UFL or FFL resulted in significant attenuation of the serum endotoxin level (*P<*0.05). A similar type of response was also shown by the animals of the HFD+LPS group when they were co-treated with colostrum. Thus, the results suggest potent endotoxin scavenging and anti-inflammatory activities of UFL and FFL formulations.

### UFL and FFL Attenuated LPS-induced Damage in Intestinal Epithelial Barrier Function both in *in vitro* and *in vivo*

**Figure 3:**
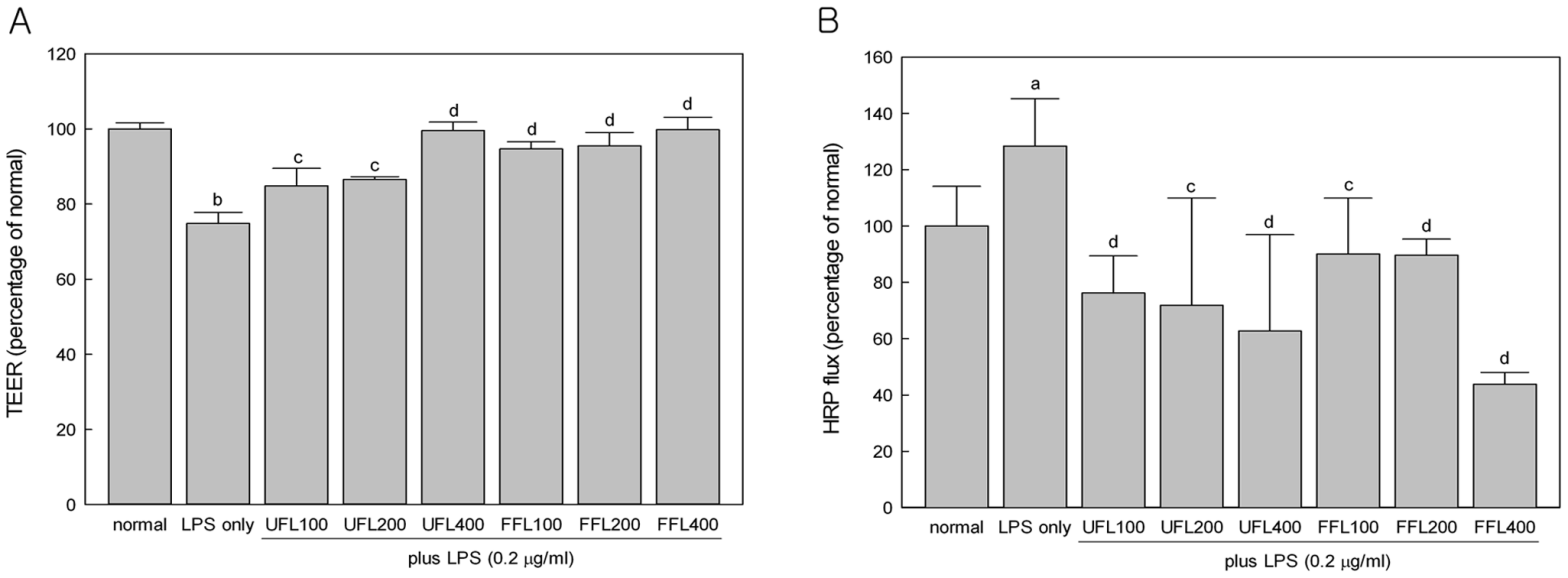
UFL and FFL treatment reduce the elevation of membrane permeability *in vitro*. (A) Immediately after pretreatment with UFL or FFL at 0 (normal, received McCoy’s 5A medium instead of herbal extraxt), 100, 200, 400 µg/ml doses for 24 h, HCT-116 cells were exposed to McCoy’s 5A medium (normal and LPS-alone) or 10 µg/ml LPS for 24 h following which measurement of transepithelial electrical residence (TEER) was performed as described in Materials and Methods section. (B) Immediately after pretreatment with UFL or FFL at 0 (normal, received McCoy’s 5A medium instead of herbal extract), 100, 200, 400 µg/ml doses for 24 h, HCT-116 cells cells were exposed to McCoy’s 5A medium (normal and LPS-alone) or 10 µg/ml LPS for 24 h following which horseradish peroxidase (HRP) flux assay was performed as described in Materials and Methods section. The experimental results in both cases are expressed as a percentage of the normal.^ a^ *P*<0.05, ^b^ *P*<0.01 compared to the normal and^ c^ *P*<0.05, ^d^ *P*<0.01 compared to the LPS alone.

Treatment with LPS resulted in significantly decreased TEER and augmented HRP flux in the *in vitro* model of intestinal epithelium (*P*<0.01) (Fig. 3A and B), indicating an impairment in the epithelial barrier function. However, co-treatment of LPS-induced cells with either UFL or FFL resulted in significant normalization of the above mentioned changes (*P*<0.01) (Fig. 3A and B). Similarly, in our *in vivo* study, feeding of rats with HFD resulted in significantly increased gut permeability (*P*<0.01) (Fig. 2A), as reflected by the elevated lactulose/mannitol (L/M) ratio in urine, while exposure of HFD-fed rats to LPS further increased the gut permeability in a significant manner (*P*<0.05). However, co-treatment with either UFL or FFL, but not colostrum, resulted in marked attenuation of the L/M ratio in the LPS control group (*P*<0.05) (Fig. 2A).

**Figure 4:**
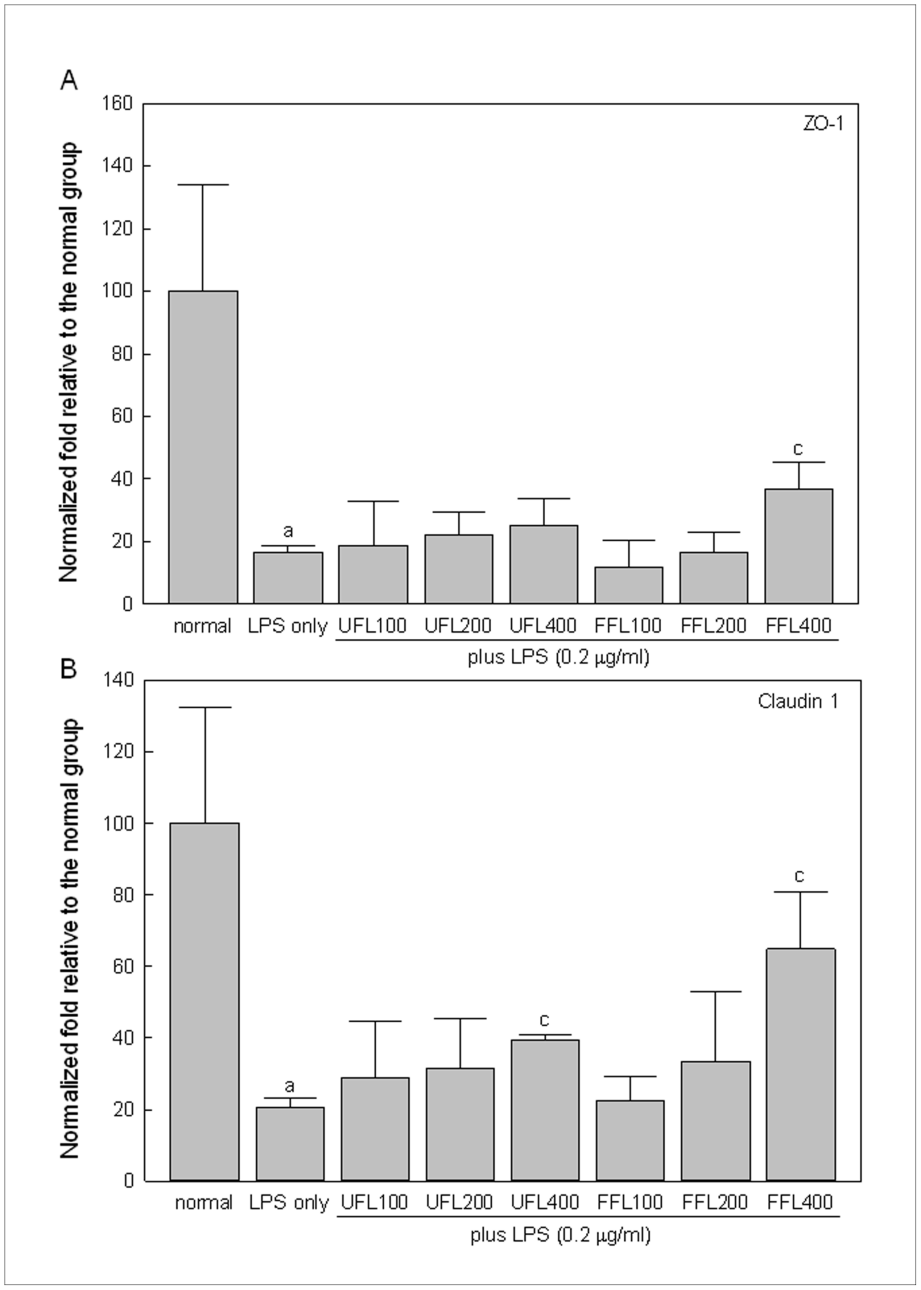
UFL and FFL positively regulate tight junction protein genes expression in HCT 116 cells. Immediately after pretreatment with UFL or FFL at 0 (normal, received McCoy’s 5A medium instead of herbal extraxt), 100, 200, 400 µg/ml doses for 2 h, HCT-116 cells were exposed to McCoy’s 5A medium (normal and LPS-alone) or 10 µg/ml LPS for 6 h following which expression of claudin-1 and zonula occludens-1 (ZO-1) genes was determined as described in Materials and Methods section. The experimental results are expressed as normalized fold change relative to the normal group. ^a^ *P*<0.05 compared to the normal and^ c^ *P*<0.05 compared to the LPS treatment alone.

In keeping with above results, our study using *in vitro* model of intestinal epithelium showed that the gene expression of claudin-1 and ZO-1, the two important members of the tight junction protein (TJP) family, was remarkably down-regulated in response to treatment with LPS (*P*<0.05, Fig. 4A and B). However, co-exposure of LPS-treated epithelial cells to the highest experimental dose of both UFL and FFL (400 µg/ml) resulted in significantly elevated expression of the claudin-1 gene (*P<*0.05) (Fig. 4A), while co-treatment of LPS–induced epithelial cells with FFL only at a dose of 400 µg/ml, but not any experimental dose of UFL, resulted in markedly up-regulated expression of the ZO-1 gene (*P*<0.05) (Fig. 4B).

### UFL and FFL Attenuated Body and Fat Mass of the HFD+LPS Group

Feeding of rats with HFD resulted in significantly augmentation of body mass and weight of abdominal and epididymal adipose tissues (*P*<0.01 versus normal group). However, treatment of HFD-fed animals with LPS did not result in any significant change in the above mentioned parameters. On the other hand, co-exposure of the HFD+LPS group to either UFL or FFL resulted in markedly decreased body mass and abdominal adipose tissue weight (*P<*0.05 or 0.01), while co-treatment of the HFD+LPS group with FFL, but not UFL, resulted in significantly decreased epididymal adipose tissue weight (*P<*0.05) (Table 3). Notably, co-exposure to colostrum also resulted in significantly attenuated body and fat mass in the HFD+LPS group (*P*<0.05, *P*<0.01).

**Table 3:** Body/adipose tissue weights and serum biochemistry parameters.

High Fat Diet	–	+	+	+	+	+
LPS (0.75 mg/kg)	–	–	+	+	+	+
Groups	normal	HFD control	LPS control	UFL	FFL	colostrum
Body mass (g)	355±27	465±33^##^	457±18^$$^	421±15^*,&&^	359±26^**,&&^	398±59^*,&^
Abdominal fat (g)	6.6±1.1	22.9±4.0^##^	26.0±2.6^$$^	15.7±3.1^*,&&^	12.7±3.7^*,&&^	13.0±4.2^*,&&^
Rela. of Abf (%)	1.87±0.32	4.97±0.84^##^	5.75±0.71^$$^	3.08±1.66^*,&&^	3.51±0.84^*,&&^	3.23±0.64^*,&&^
Epididymal fat (g)	5.38±0.87	13.39±1.81^##^	14.77±2.92^$$^	12.07±1.59	8.96±1.37^*,&^	9.51±3.03^*,&^
Rela. of Epf (%)	1.52±1.26	2.89±0.27^##^	3.27±0.56^$$^	2.75±0.18	2.41±0.26^*,&^	2.27±0.54^*,&^
T. chol (mg/dl)	46.1±4.9	67.1±14.7^#^	78.8±16.7^$$^	61.8±7.4^&^	54.5±7.5^&&^	65.6±11.1
HDL (mg/dl)	38.2±4.9	29.6±5.9^#^	21.5±4.4*^,$$^	23.8±3.7*	30.2±6.4^&^	25.2±5.7
TG (mg/dl)	28.1±7.8	42.1±12.7^#^	78.2±33.3^*,$^	55.8±9.9	41.5±6.7^&^	42.7±17.8^&^
AST (IU/l)	27.2±9.3	50.4±25.6^#^	124.4±63.4*^,$$^	13.5±4.2**^,&&^	11.7±3.1**^,&&^	13.2±1.6**^,&&^

Abbreviations: Rela. Of Abf, relative weight of abdominal fat; Rela. of Epf, relative weight of epididymal fat; T. chol, total cholesterol; HDL, high density lipoprotein; TG: triglyceride. Body weights were measured after 18 h LPS injection before sacrifice, and relative organ weight was calculated as fat weight/body weight. Data were shown as mean ± SD; ^#^ *P<*0.05, ^##^ *P<*0.01, compared to the normal group; ^*^ *P<*0.05, ^**^ *P<*0.01 compared to the HFD control group; ^$^ *P<*0.05, ^$$^ *P<*0.01, compared to the normal group; ^&^ *P<*0.05, ^&&^ *P<*0.01 compared to the LPS control group (n = 8).

### UFL and FFL Ameliorated Serum Lipid Parameters of the HFD+LPS Group

Feeding of animals with HFD resulted in marked elevation of the level of serum total cholesterol (TC), TG and AST activity, and reduced the serum HDL content (*P*<0.05), while treatment of HFD-fed animals with LPS resulted in a further increase in the serum TG level and AST activity along with a decrease in the serum HDL content (*P*<0.05) (Table 3). Co-treatment of the HFD+LPS group with both UFL and FFL resulted in marked attenuation of the serum AST activity (*P*<0.01). However, co-exposure of the HFD +LPS group to FFL, but not UFL, resulted in significant attenuation of the serum TG level and augmentation of the serum HDL content (*P*<0.05). In contrast, serum TC level of the HFD +LPS group was reduced by both UFL and FFL co-treatments, but more effectively by FFL (*P<*0.01) compared to UFL (*P*<0.05). Notably, similar to FFL, colostrum co-treatment in the HFD+LPS group also resulted in significant attenuation of the serum TG level and AST activity. However, colostrum did not produce any significant change in the serum levels of TC and HDL in the HFD+LPS group.

### UFL and FFL Attenuated HFD and LPS-induced Accumulation of Lipid Droplets and Inflammatory Cellular Infiltration in the Liver

**Figure 5:**
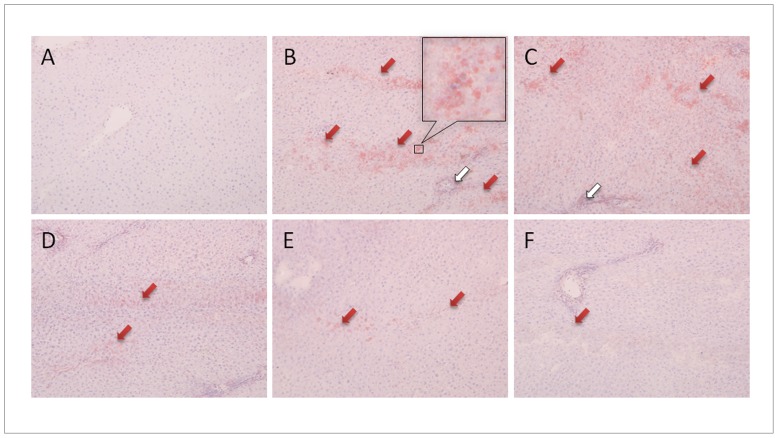
UFL and FFL attenuate HFD and HFD+LPS-induced inflammation and obesity-related histological changes in liver. Following the termination of experimental schedule, the animals were sacrificed and livers were collected and stored in −70°C. After embedding and making frozen sections of the tissues, the slides were stained with Oil red O solution and then counter-stained with hematoxylin. The pathophysiological examination of the tissue sections was performed under light microscopy with 400×magnification as described in Materials and Methods section. (A): normal, (B): HFD control, (C): LPS control, (D): HFD+LPS+UFL, (E): HFD+LPS+FFL, (F): HFD+LPS+colostrum. The red arrowheads indicate lipid droplets stained by Oil red O, and the white arrowheads indicate the infiltration of inflammatory cells.

Histopathological observation of the liver revealed the deposition of a large number of lipid droplets (red arrowheads) in the HFD group, indicating the state of fatty liver (Fig. 5). In addition, a sign of infiltration of inflammatory cells (white arrowheads) in hepatic tissue was also evident in the same group. Similar histopathological changes were also observed in the hepatic tissue of the HFD+LPS group; however, co-treatment of the same group with both UFL and FFL evidently alleviated these changes. Likewise, colostrum co-treatment also resulted in markedly ameliorated accumulation of lipid droplets and inflammation in the liver of the HFD+LPS group.

### UFL and FFL Depleted the Vital Inflammatory Markers in the Rat Serum

**Figure 6:**
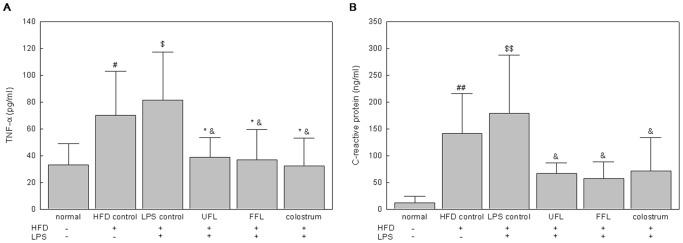
UFL and FFL diminish the vital inflammatory markers in the serum of HFD-fed plus LPS-treated rats. After the termination of experimental schedule, the blood was collected from the animals following which the serum endotoxin level was determined as described in Materials and Methods section. ^#^ *P*<0.05, ^##^ *P*<0.01, compared to the normal group; ^*^ *P*<0.05 compared to the HFD control group; ^$^ *P*<0.05, ^$$^ *P*<0.01, compared to the normal group; ^&^ *P*<0.05, ^&&^ *P*<0.01 compared to the LPS control group (n = 8).

Exposure of the animals to HFD and HFD plus LPS resulted in a significant increase in the content of TNF-α and CRP in serum, as compared with the normal group (Fig. 6A and B, respectively, *P<*0.05 or 0.01). Nevertheless, both UFL and FFL co-treatments in HFD+LPS group provoked a marked reduction in the levels of these inflammatory markers as compared with the HFD+LPS group alone (*P<*0.05).

### UFL and FFL Modified the Distribution of Gut Microbiota

**Figure 7:**
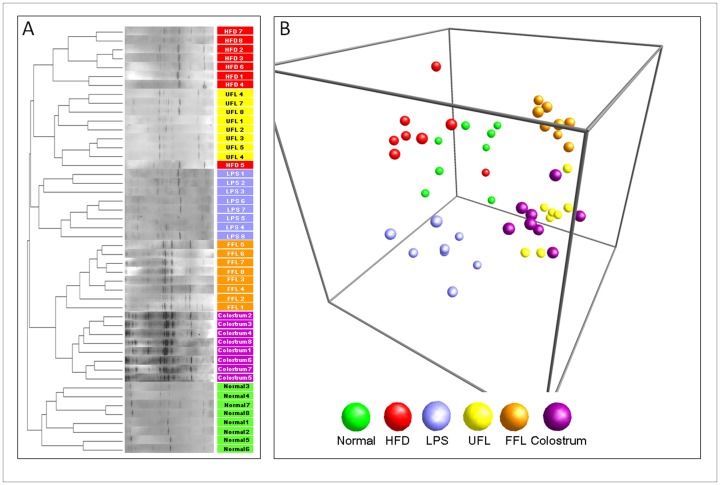
PCR-denaturing gradient gel electrophoresis (PCR-DGGE) fingerprinting and principal coordinate analysis (PCA) of rat fecal samples. (A) Following the termination of treatment schedule, the animals were fasted for 12 h with access to water *ad libitum*. After another 20 h of fasting, the stool samples were collected following which the fecal microbial communities were analyzed by DGGE as described in Materials and Methods section. (B) PCA of the data was performed based on distance matrix (two-dimensional array) to further evaluate the similarity between bacterial communities.

Co-treatment with UFL or FFL induced an obvious change in the microbial community in contrast with the normal and control groups, as shown by the distinct clustering in the DGGE profile and PCA analysis (Fig. 7A and B). This change was also found in the colostrum treated group.

**Figure 8:**
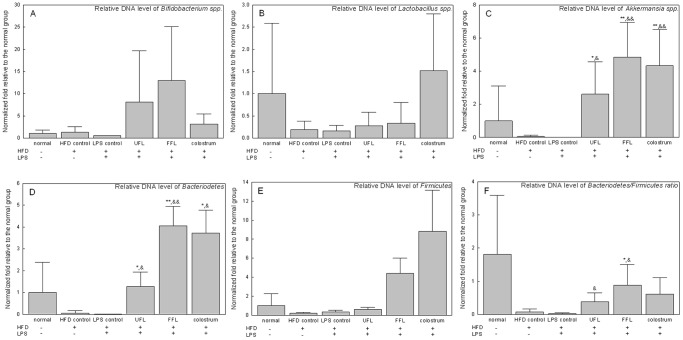
The relative DNA (gene-encoding 16S rRNA) content of related microbes in rat fecal samples. Following the termination of treatment schedule, the animals were fasted for 12*ad libitum*. After another 20 h of fasting, the stool samples were collected following which the abundance of 16S rRNA gene of the bacterial strains were determined as described in Materials and Methods section. The results are expressed as normalized fold values relative to the normal group. ^*^ *P*<0.05,^ **^ *P*<0.01 compared to the HFD control group; ^&^ *P*<0.05, ^&&^ *P*<0.01 compared to the LPS control group (n = 8).

Treatment with HFD and HFD plus LPS resulted in considerable lowering of the relative abundance of *Akkermansia muciniphila*, Bacteriodetes, Firmicutes, and the Bacteroidetes/Firmicutes ratio, as compared with the normal group. Conversely, *Akkermansia muciniphila*, Bacteroidetes and the Bacteroidetes/Firmicutes ratio were significantly higher in the UFL or FFL treated group (*P<*0.05 or 0.01) (Fig. 8C, D and F). Although co-treatment with UFL or FFL altered the relative abundance of *Lactobacillus spp*., *Bifdobacterium spp*., (Fig. 8A and B) and *Ruminococcus spp.* (data not shown), no statistical significance was observed, as compared with the control groups. Colostrum treatment also showed a pattern similar to that with UFL and FFL treatment.

## Discussion

In general, commensal microflora reside in the digestive tract of mammals throughout the life, especially in the intestinal lumen with a large population [43]. Among them, the Gram-negative bacteria constantly generate LPS as an integral component of their outer cell membrane [44]. Therefore, it is conceivable that intestinal lumen can play a vital role as an endogenous source of LPS in mammalian system [45]. LPS is considered as one of the key contributing factor for systemic inflammatory responses [28] as it has inherent property to induce macrophages to generate pathophysiological factors, such as inflammatory cytokines (TNF-α, IL-6 and COX-2 etc.), free radicals, such as NO and critical proteins related with bacterial infection and inflammation, such as CRP [46]–[48]. Therefore, amelioration of LPS-induced inflammatory responses can be considered as one of the rational therapeutic approaches to combat inflammation and inflammation-related metabolic disorders. In the present study, both UFL and FFL exhibited marked *in vitro* anti-inflammatory activities, as evidenced by suppression of LPS-induced production of NO and down regulation of the LPS-triggered expression of TNF-α, IL-6 and COX-2 genes in RAW 264.7 cells (Fig. 1). In keeping with this, our *in vivo* results revealed that both UFL and FFL inhibited the serum TNF-α and CRP levels in a HFD-fed rat model being exposed to LPS insult (Fig. 6). Accordingly, based on these results, it is conceivable that both UFL and FFL possess potent anti-inflammatory activities that are operative against LPS-induced insult.

It has been found that the exposure to LPS may directly or indirectly cause destabilization of intestinal barrier function leading to an augmentation of gut permeability [12], [49]. This is in agreement with our *in vitro* study suggesting endotoxin-mediated insult is associated with impairment of the barrier function of intestinal epithelium as evident by a marked depletion of TEER and a significant augmentation of HRP flux in HCT-116 cells in response LPS treatment (Fig. 3A and B). It has been surmised that increased intestinal permeability can further boost endotoxemia which in turn may trigger inflammation and metabolic disorders [12]. However, both UFL and FFL at all experimental doses enhanced TEER and depleted HRP flux significantly in the LPS-treated cells, implying beneficial impact of these two formulations on the permeability of intestinal epithelial cells under endotoxemia. Our *in vitro* study further demonstrates that LPS insult to HCT-116 cells is associated with reduction in the gene expression of ZO-1 and claudin-1, the two vital tight junction proteins being involved in controlling intestinal epithelial permeability, in agreement with previous reports [50], [51]. However, FFL at 400 µg/ml dose markedly increased the gene expression of ZO-1, while both UFL and FFL at 400 µg/ml dose significantly increased the gene expression of claudin-1 in the LPS-treated cells, in keeping with their ability to ameliorate LPS-mediated impairment of membrane permeability as mentioned above.

It has been also demonstrated that feeding of animals with HFD alters the gut microflora composition [52] and that the modulation of gut microbiota is associated with an increased intestinal permeability that eventually leads to the development of metabolic endotoxemia, inflammation, and metabolic disorders [12]. This is in keeping with our *in vivo* study, where the urine L/M ratio, a valuable detection parameter of gut permeability, [45] was significantly increased in parallel with a significant augmentation in serum endotoxin level in rats in response to HFD feeding (Fig. 2A and B). Furthermore, a dramatic increase in both intestinal permeability and serum endotoxin level in HFD-fed animals due to exposure to exogenous LPS suggests the additive effect of this agent to the prevailed condition of endotoxemia. It has been surmised that an increase in luminal LPS due to altered gut microbiota can augment toll-like receptor 4 (TLR4) activation at the epithelium, leading to altered tight junction permeability [53]. Taking together and considering our *in vitro* results, it is conceivable that an inhibition in gene expression of ZO-1 and claudin-1 by endotoxin might account at least in part for the decreased intestinal permeability in the animals of HFD and HFD+LPS groups. This is in agreement with previous study suggesting that HFD triggers metabolic changes that impair gut barrier function, as reflected both by the decrease in TEER and gene expression of ZO-1 in the proximal colon [54]. Similarly, an increase in whole-gut permeability in HFD-fed mice was found to be associated with a reduction in gene expression of tight junction proteins including ZO-1 [12]. However, most importantly, co-treatment of HFD+LPS group with UFL or FFL in our experiments markedly decreased the serum endotoxin level and reduced the urine L/M ratio to very nearly to the normal level (Fig. 2A and B). Taking the above *in vivo-in vitro* correlated results together, it is conceivable that UFL and FFL exert a beneficial effect on gut permeability of HFD+LPS group probably via depletion of serum endotoxin level as well as protection of the intestinal epithelial cells from endotoxin-insult through their anti-inflammatory activities, and amelioration of the expression of vital tight junction proteins in the epithelium. In this respect, in gross, FFL appeared to be slightly more potent than UFL in exerting beneficial impact on the membrane permeability of intestinal epithelial cells.

**Figure 9:**
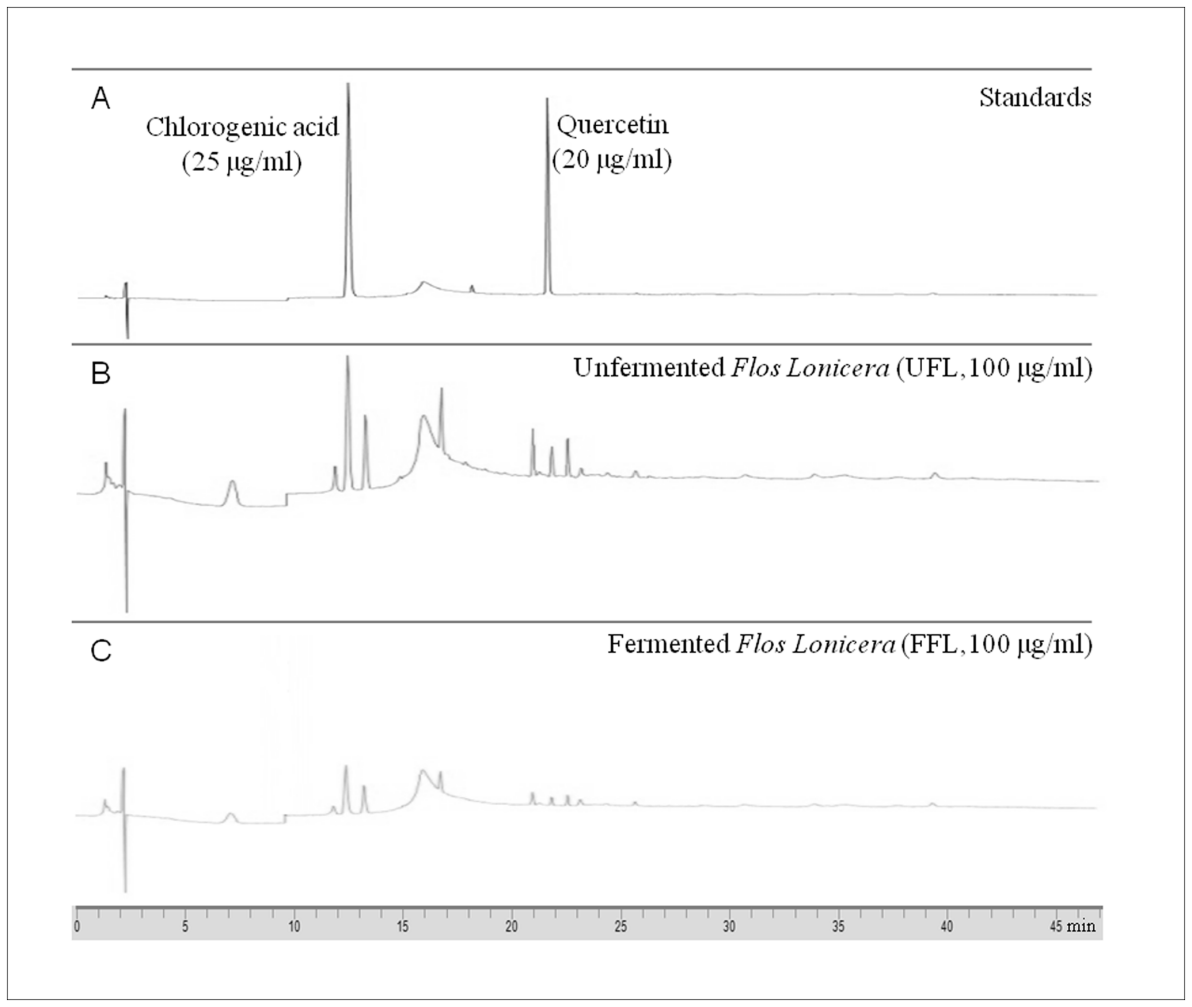
HPLC fingerprint of UFL and FFL. The HPLC elution profile of extracted samples of unfermented and fermented Flos Lonicera and standards (chlorogenic acid and quercetin). The extracted samples and standards were filtrated and subjected to HPLC analysis as described in Materials and Methods section. A: standard: Chlorogenic acid (left) 25 µg/ml and Quercetin (right) 20 µg/ml, B: UFL (Unfermented *Flos Lonicera*) 100 µg/mL, C: FFL (fermented *Flos Lonicera*) 100 µg/mL. All chromatograms were produced at a wavelength of 238 nm.

In our *in vivo* study, long-term (eight weeks) feeding of animals with HFD induced severe obesity, as reflected by a significant increase in body weight, abdominal and epididymal fat weight, and serum levels of total cholesterol, TG and AST as well as a significant reduction of serum HDL, accompanied with deposition of lipid droplets in the liver (Fig. 5). Administration of a single dose of LPS (0.75 mg/kg) in HFD-fed animals further imposes the obesity impact, as evidenced by a significantly higher levels of serum AST and TG and a significantly lower level of serum HDL in HFD+LPS group compared to HFD control group (Table 3). Collectively, these observations suggest an induction of metabolic dysfunction in animals as a consequence of HFD or HFD+LPS treatments. The role of LPS in the development and onset of metabolic diseases has been well documented [55]. More specifically, it has been found that continuous low-grade endotoxemia, a common phenomenon in LPS-induced insult, is a potent contributor of obesity and related metabolic disorders [14]. The infusion of LPS into wild type mice for a period of 4 weeks was found to increase whole body, liver, and adipose tissue weights, as well as adipose and liver inflammation (such as elevated TNF-α, IL-1, IL-6, and plasminogen activator inhibitor-1 [11]). Importantly, the treatment of HFD+LPS group with UFL or FFL in our experiments resulted in significant reduction in body and fat mass and marked attenuation of the serum levels of total cholesterol and AST. However, treatment of HFD+LPS group with FFL, but not with UFL, caused significant decrease in the TG level and restoration of the HDL level. The anti-obesity impact of UFL and FFL formulations was also evident in our histopathological finding, where both of the formulation caused a marked depletion of the lipid droplets in the hepatic tissue of the animals of HFD+LPS group (Fig. 5). Thus taking the above results together, it is conceivable that both UFL and FFL can exert beneficial impact against metabolic endotoxemia and obesity. However, in this respect, FFL was found to be more effective than UFL to a certain extent, although, no definite conclusion can be made on the mechanism behind this as our HPLC fingerprinting analyses have revealed no significant difference between the major ingredients of these two formulations (Fig. 9). One possible explanation is the expected probiotic effect of *Lactobacillus plantarum* which were used for fermentation of the *Lonicera* extract. Some probiotics are shown to exert anti-obesity impact by regulating lipid and glucose metabolism [56], [57], generating conjugated linoleic acid [58], [59], decreasing the size of adipocytes and augmenting the number of small adipocytes in white adipose tissue [60], and regulating leptin expression [61].

The mammalian gastrointestinal tract is colonized by trillions of microbes, which are known to produce beneficial impact in the host, including the promotion of digestion and absorption, regulation of intestinal permeability, and prevention of tumor formation [62]–[64]. The studies accompanied with sequencing-based approaches have revealed that the human gut microbiota encompasses more than 1000 phylotypes [55]. Among the intestinal microbial population, 99% of microflora primarily originate from five types of phyla: Firmicutes, Bacteroidetes, Actinobacteria, Fusobacteria, and Proteobacteria [51] and it has been estimated that Bacteroidetes and Firmicutes account for >90% of the total gut microbiota [55]. Substantial evidence indicates that gut microbiota play a significant role in the development of obesity, obesity-associated inflammation and cardiometabolic complications [55]. Furthermore, using diet-induced obese animal model, it has been proposed that HFD-induced obesity is associated with changes in the gut microbiota and gut inflammation [53]. Previous studies have also suggested that the imbalance in the ratio of intestinal bacteria plays a vital role in the development of obesity which is driven through a number of factors, such as promotion of energy harvest from diet, induction of systemic inflammation, and acceleration of fat deposition [7], [65]. In the current study, the cluster analysis of PCR-DGGE fingerprinting indicated that treatment with both herbal formulations resulted in noticeable changes in the pattern of the bacterial community (Fig. 7A) especially, when compared with the HFD and LPS control groups. Additionally, PCA analyses of DGGE fingerprints also indicated the separate clusters among each group (Fig. 7B). Notably, the clustering of the FFL group and colostrum group were more similar in comparison with other groups. These findings imply that FFL exerts probiotic efficacy, which is analogous with colostrum [66].

**Figure 10:**
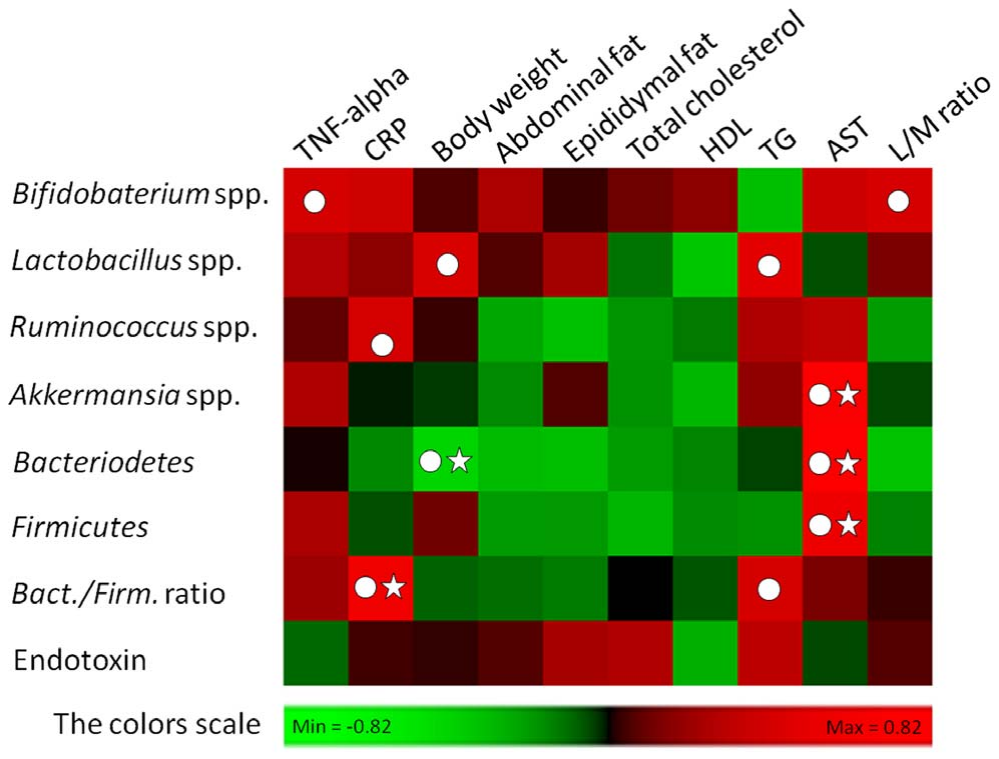
Analyses of correlation between gut flora composition and host metabolic parameters. The entire data of all experimental groups except normal were gathered and analyzed by SPSS software (17.0 version) using two-tailed Pearson’s correlation test. The scores of Pearson’s correlation were figured by PermutMatrix software (Version 1.9.3 EN) using heatmap plots. As the colors scale shown, green color indicates a positive correlation, while red color shows a negative correlation. (A symbol of ○ indicates absolute value of Pearson r >0.4; a symbol of ☆ indicates statistical significance of *P*<0.05).

Other ample evidence indicates that a lower Bacteroidetes/Firmicutes ratio (B/F ratio), which reflects phylum-wide increase in Firmicutes and/or reduction in Bacteroidetes [8], [64], [67], [68], is linked to obesity [55]. In our study, treatment with UFL and FFL resulted in a considerable increase in the relative abundance of Bacteriodetes, Firmicutes and the Bacteroidetes/Firmicutes ratio, as compared to the HFD or LPS control groups. However, according to the result of interrelationship between gut microbiota composition and host metabolic parameters, Bacteroidetes showed a significant negative correlation (*P*>0.05 and Pearson r<−0.4) with body weight rather than other gut commensal bacteria (Fig. 10). Although Ganesh et al. reported that *Akkermansia muciniphila* exacerbates *Salmonella typhimurium* induced gut inflammation in animal model [69], recently two studies have reported that *Akkermansia muciniphila* ameliorates high fat diet induced obesity, metabolic endotoxemia and type 2 diabetes via modulation of Foxp3 regulatory T cells [10], [70]. Notably, our result (Fig. 8C) revealed that the intestinal population of *Akkermansia muciniphila* was markedly enhanced by UFL and FFL, as compared to the HFD and LPS control groups. Based on this, it is conceivable that in our study, *Akkermansia muciniphila* could play an essential role in ameliorating HFD or HFD+LPS induced obesity and endotoximia.

Taken together, our study highlights that both unfermented and fermented *Flos Lonicera* formulations could significantly improve HFD-induced obesity and related endotoxemia. Our study also suggests that modulation in the distribution of the intestinal flora, especially restoration of relative abundance of *Akkermansia muciniphila* and the B/F ratio by the above two mentioned formulations could play an essential role in combating HFD and HFD+LPS induced enhancement in gut permeability, development of endotoximia, and inflammation, where in some cases, FFL produced better effects compared to UFL.
